# Identification of the Transcription Factor Relationships Associated with Androgen Deprivation Therapy Response and Metastatic Progression in Prostate Cancer

**DOI:** 10.3390/cancers10100379

**Published:** 2018-10-11

**Authors:** Nitya V. Sharma, Kathryn L. Pellegrini, Veronique Ouellet, Felipe O. Giuste, Selvi Ramalingam, Kenneth Watanabe, Eloise Adam-Granger, Lucresse Fossouo, Sungyong You, Michael R. Freeman, Paula Vertino, Karen Conneely, Adeboye O. Osunkoya, Dominique Trudel, Anne-Marie Mes-Masson, John A. Petros, Fred Saad, Carlos S. Moreno

**Affiliations:** 1 Genetics and Molecular Biology PhD Program, Emory University, Atlanta 30322, Georgia; nvsharm@emory.edu; 2 Department of Urology, Emory University School of Medicine, Atlanta 30322, Georgia; kathryn.pellegrini@emory.edu (K.L.P.); adeboye.osunkoya@emory.edu (A.O.O.); jpetros@emory.edu (J.A.P.); 3 Centre de Recherche du Centre Hospitalier de l’Université de Montréal (CRCHUM)/Institut du cancer de Montréal, Montreal, QC H2X 0A9, Canada; veronique.ouellet.chum@ssss.gouv.qc.ca (V.O.); oxalique@live.com (E.A.-G.); lufossouo@yahoo.ca (L.F.); dominique.trudel.chum@ssss.gouv.qc.ca (D.T.); anne-marie.mes-masson@umontreal.ca (A.-M.M.-M.); fredsaad@videotron.ca (F.S.); 4 MSTP/MD PhD, Emory University, Atlanta 30322, Georgia; fgiuste@emory.edu; 5 Pathology and Laboratory Medicine, Emory University School of Medicine, Atlanta 30322, Georgia; selkorner@yahoo.com (S.R.); kenneth.a.watanabe@emory.edu (K.W.); 6 Division of Cancer Biology and Therapeutics, Departments of Surgery & Biomedical Sciences, Samuel Oschin Comprehensive Cancer Institute, Cedars-Sinai Medical Center, Los Angeles, CA 90048, USA; sungyong.you@cshs.org (S.Y.); michael.freeman@cshs.org (M.R.F.); 7 Department of Radiation Oncology, Emory University School of Medicine, Atlanta 30322, Georgia; pvertin@emory.edu; 8 Winship Cancer Institute of Emory University, Atlanta 30322, Georgia; kconnee@emory.edu; 9 Department of Human Genetics, Emory University, Atlanta 30322, Georgia; 10 Atlanta VA Medical Center, Decatur, GA 30033, USA; 11 Department of Pathology and Cellular Biology, Université de Montréal, Montreal, QC H3C 3J7, Canada; 12 Department of Medicine, Université de Montréal, Montreal, QC H3C 3J7, Canada; 13 Department of Surgery, Université de Montréal, Montreal, QC H3C 3J7, Canada; 14 Université de Montréal Endowed Chair in Prostate Cancer, Montreal, QC H3C 3J7, Canada

**Keywords:** androgen deprivation therapy, prostate cancer, transcriptional networks, metastasis

## Abstract

Background: Patients with locally advanced or recurrent prostate cancer typically undergo androgen deprivation therapy (ADT), but the benefits are often short-lived and the responses variable. ADT failure results in castration-resistant prostate cancer (CRPC), which inevitably leads to metastasis. We hypothesized that differences in tumor transcriptional programs may reflect differential responses to ADT and subsequent metastasis. Results: We performed whole transcriptome analysis of 20 patient-matched Pre-ADT biopsies and 20 Post-ADT prostatectomy specimens, and identified two subgroups of patients (high impact and low impact groups) that exhibited distinct transcriptional changes in response to ADT. We found that all patients lost the AR-dependent subtype (PCS2) transcriptional signatures. The high impact group maintained the more aggressive subtype (PCS1) signal, while the low impact group more resembled an AR-suppressed (PCS3) subtype. Computational analyses identified transcription factor coordinated groups (TFCGs) enriched in the high impact group network. Leveraging a large public dataset of over 800 metastatic and primary samples, we identified 33 TFCGs in common between the high impact group and metastatic lesions, including SOX4/FOXA2/GATA4, and a TFCG containing JUN, JUNB, JUND, FOS, FOSB, and FOSL1. The majority of metastatic TFCGs were subsets of larger TFCGs in the high impact group network, suggesting a refinement of critical TFCGs in prostate cancer progression. Conclusions: We have identified TFCGs associated with pronounced initial transcriptional response to ADT, aggressive signatures, and metastasis. Our findings suggest multiple new hypotheses that could lead to novel combination therapies to prevent the development of CRPC following ADT.

## 1. Introduction

Prostate cancer is one of the most commonly diagnosed cancers and the second leading cause of cancer death in men in the United States [1]. Currently, androgen deprivation therapy (ADT) is one component of care for patients with locally advanced prostate cancer, and advanced or metastatic prostate cancer [2,3]. Patients with advanced and metastatic prostate cancer will usually respond favorably initially, but will frequently experience disease progression despite the therapy [4]. This type of cancer is termed castration-resistant prostate cancer (CRPC) and is typically associated with metastatic disease and a poor prognosis, rendering it virtually incurable [5]. However, there is a subset of patients with locally advanced prostate cancer who benefit from ADT, in conjunction with other treatments such as radiation therapy, and experience improved disease-free and overall survival [3]. In these patients, ADT forces changes in the tumor biology that result in distinct molecular profiles. While many studies focus on the dysregulated gene expression programs characteristic of ADT response, the upstream regulators that characterize these differential transcriptional programs have not been comprehensively elucidated [6].

Androgen receptor (AR) stimulation and downstream signaling is critical for the initiation and progression of prostate cancer [7]. Upon androgen ligand activation, AR can function as a transcription factor to regulate target genes. AR-signaling is reestablished in CRPC despite the initial inhibition by ADT, due to mutational adaptations of the AR gene, including gene amplification and the expression of alternative AR splice variants (AR-V) [8,9]. Interestingly, DNA binding and the subsequent gene expression profiles may vary between full length AR (AR-FL) and an ARV [10]. Stimulation of AR transcriptional activity was found to be largely dependent on the cooperation with specific co-activators [11,12,13]. Moreover, AR transcriptional activity can be inhibited via targeting of specific co-activators [14]. Additionally, other AR-independent signal transduction pathways can be aberrantly activated by facilitating crosstalk with, and/or bypass of, the AR-signaling pathway. Many studies have identified actionable signaling pathways, including the Wnt and PI3K-AKT-PTEN signaling pathways, as significantly altered in tumors from patients with metastatic CRPC, as compared to hormone naïve localized tumors [15,16]. Thus, understanding the key regulators responsible for the progression to metastasis in an androgen-deprived environment is essential.

Here we identify novel putative transcription factor coordinated groups (TFCGs) that characterize the differential transcriptional signatures in tumors of patients who received ADT, as well as the progression from localized prostate cancer to a metastatic disease. We generated whole transcriptome gene expression data from 20 patient-matched formalin-fixed prostate paraffin embedded (FFPE) needle core biopsies, taken before initiation of neoadjuvant ADT (pre-ADT Bxs), and the corresponding FFPE radical prostatectomy samples, acquired after ADT (post-ADT RPs), and leveraged a large dataset (*n* > 800) of multiple publicly available cohorts of primary and metastatic tumors [17]. We integrated the protein-protein interaction, gene expression, and DNA binding data by utilizing the PANDA (Passing Attributes between Networks for Data Assimilation) method [18,19] to infer condition-specific relationships between the transcription factors and putative gene targets. We combined these analyses to uncover groups of putative transcription factor regulators that were unique to patients demonstrating a strong transcriptional response to ADT, and to patients with metastatic prostate cancer. Our analysis leverages multiple datatypes and independent datasets to find common TFCGs that may serve as putative regulators of prostate cancer aggressiveness.

## 2. Results

### 2.1. Differential Expression Analysis Reveals Two Distinct Transcriptional Responses to ADT

The total RNA was derived from matched pairs of pre-ADT and post-ADT samples from 20 patients. All patients received neoadjuvant ADT and one patient also underwent radiation therapy. The median ADT duration time between the biopsy and RP was 3 months, with a range of 1 to 8 months (Supplementary Table S1, column W). ADT treatment regimens are indicated in Supplementary Table S1, column T. Six of the patients developed metastasis (ranging 42–166 months after RP, Supplementary Table S1, column BB). The samples included in the study were comprised of 20 matched needle core biopsies obtained before ADT, and 20 radical prostatectomies obtained after ADT. On average, we obtained 91 M reads per sample, with a 64× coverage of the transcriptome.

**Figure 1:**
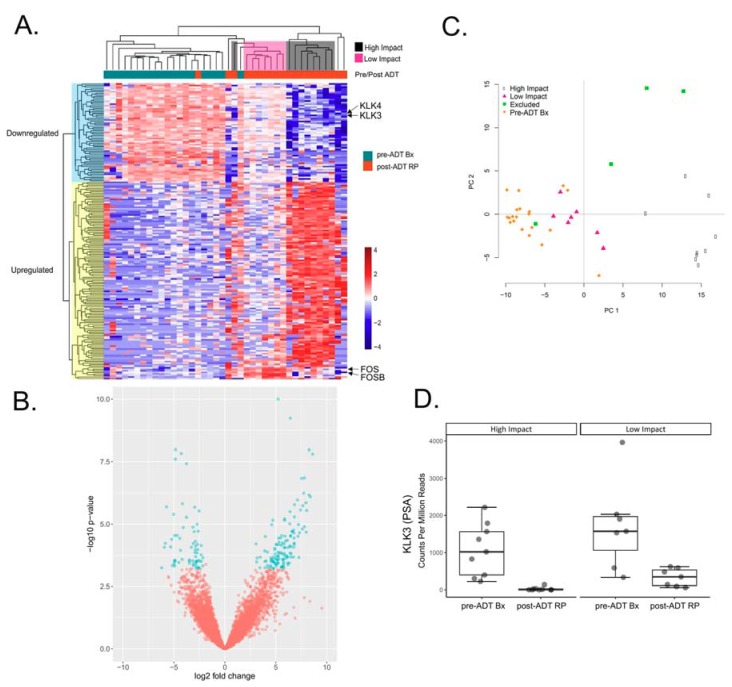
The hierarchical clustering and principal component analysis (PCA) of 190 significantly differentially expressed genes in 20 matched Pre-ADT biopsies and Post-ADT radical prostatectomies (RPs). (**A**) Clustering reveals two groups of Post-ADT RPs displaying segregated based on the expression of upregulated and downregulated genes (high- or low-impact groups, respectively); (**B**) Volcano plot highlighting 190 significantly differentially expressed genes; (**C**) PCA reveals 4 post-ADT RP samples as not clustering with either the high or low impact groups; (**D**) Boxplot depicting KLK3 expression in counts per million mapped reads demonstrates that the decrease in the KLK3 expression is significantly more pronounced in the high impact group than in the low impact group.

We performed a differential expression analysis of the RNA-Seq data and identified 190 significantly differentially expressed genes with a fold change greater than or equal to 2 (FDR < 0.05) (Figure 1B and Supplementary Table S2). A total of 127 of these genes were upregulated and 63 significant genes were downregulated. To gain initial insights into signaling pathways associated with all post-ADT RPs, we performed an Ingenuity Pathway Analysis (IPA) on the differentially expressed genes. As expected, we observed an enrichment of downregulated genes that are typically altered in response to agents that promote AR-signaling, such as dihydrotestosterone, and the AR agonist metribolone (R1881) (Supplementary Table S3). Additionally, we found that genes inhibited by U0126 (an inhibitor of the MAPK signaling pathway [20]), were downregulated, while genes activated by PDGF, a growth factor that stimulates MAPK signaling, were activated. Furthermore, there was an enrichment of upregulated genes within the estrogen signaling pathway despite a lack of increase in ESR1 expression (Mann–Whitney test *p*-value = 0.06). (Supplementary Table S3). These data support the expected repression of androgen-driven genes as well as possibly compensatory increases of estrogen and PDGF-MAPK signaling following ADT. 

Hierarchical clustering segregated the post-ADT RP samples based on the expression of two clusters of upregulated and downregulated genes that defined the pre- and post-ADT conditions (Figure 1A). There was a common decrease in the downregulated genes among all but one of the post-ADT RP samples, though the degree of repression was strikingly more pronounced in one group. One RP sample was clustered closely with the pre-ADT samples mainly due to the relatively higher expression of downregulated genes, discriminating it from the rest of the post-ADT RP samples. The upregulated genes further segregated the post-ADT samples based on the increased expression in one subgroup (high impact group) but relatively unchanged expression after ADT in the other subgroup (low impact group). Principal component analysis (PCA) of differentially expressed genes confirmed the observations of the hierarchical clustering. PCA revealed a distinct cluster segregating the high impact group away from the low impact group. The low impact group was more similar to the pre-ADT Bx. Three outlier RP samples did not cluster with any group, and one RP sample very closely segregated with the pre-ADT Bx both in PCA and hierarchical clustering of differentially expressed genes, denoting that this tumor remained relatively unchanged after ADT (Figure 1C).

Although there was no significant difference in the ADT exposure time in the high and low impact group (*p* < 7.18 × 10^−2^ Mann–Whitney test), we observed striking differences in the transcriptional signatures after ADT. We evaluated the differences in the relative gene expression of both the “upregulated” and “downregulated” gene sets in the pre-ADT Bx, low impact, and high impact groups (Figure 1A, Supplementary Figure S1). The mean expression of the upregulated genes in these three groups was significantly different (one-way ANOVA and Tukey Honest Significant Differences *p*-value = 2.14 × 10^−8^). The high impact group displayed over a 4.5-fold higher mean expression of “upregulated” genes as compared to the low impact group. Moreover, the mean expression of the “downregulated” genes in the pre-ADT Bx, low impact, and high impact groups were also significantly different (one-way ANOVA and Tukey Honest Significant Differences *p*-value = 3.59 × 10^−11^). The “downregulated” mean gene expression was more than 3-fold lower in the high impact group than in the low impact group (Supplementary Figure S1). For example, the KLK3 (Prostate Specific Antigen or PSA) gene expression was more significantly decreased in the high impact group than in the low impact group after ADT (*p* < 2.14 × 10^−3^ Mann–Whitney test), concordant with the IPA results suggesting a decrease in androgen driven genes (Figure 1D). To biologically characterize the transcriptional changes specific to the low and high impact groups, we employed a subtyping method developed by You et al. [17]. The prostate cancer subtypes (PCS) defined in this scheme utilize gene signatures based on a priori prostate cancer-relevant biological pathways as determined by You et al. to segregate tumors into three groups: prostate cancer subtype 1, 2, or 3 (PCS1-3) [17]. The PCS1 subtype is enriched for genes involved in androgen receptor variant (AR-V) (ligand-independent, constitutively active) activation, and is associated with a poor prognosis. The PCS2 subtype is enriched for genes indicative of AR activation and has a variable prognosis. The PCS3 subtype is characterized by the low activation of AR or AR-V associated genes (AR-suppressed) and has a variable prognosis. 

**Figure 2:**
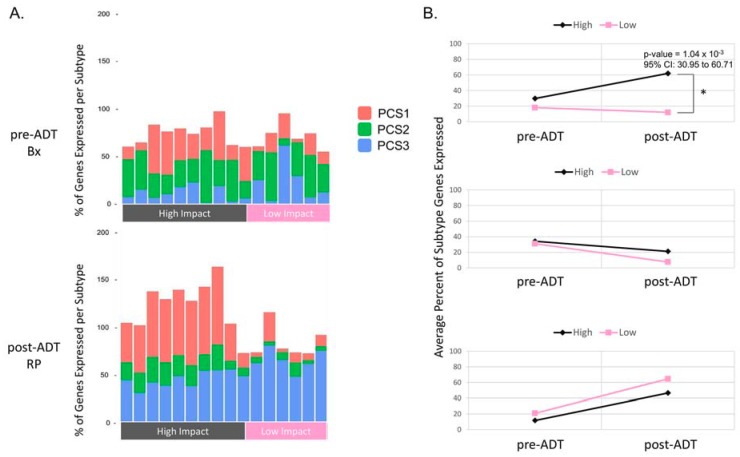
Divergent expression of the PCS1 genes in the high impact group and common loss of PCS2 and PCS3 after ADT. (**A**) The bar plots depict three stacked bars. Each bar displays the fraction of the subtype-specific genes expressed in a given subtype that is more than two-fold above the median across all samples. Both the high and low impact groups lose the expression of the PCS2 genes after ADT, but the high impact group samples display a retention and increase in the PCS1 signature after ADT, while the low impact group loses the PCS1 signature but displays an increase in the PCS2 gene expression; (**B**) Plots depict the average percent change of the subtype gene expression before and after ADT. The PCS1 gene signature is significantly higher in the high impact group after ADT than in the low impact group after ADT (*p*-value = 1.04 × 10^−3^ 95% CI: 30.95 to 60.71 by Mann–Whitney test).

To classify the relative PCS makeup of the 40-matched pre-ADT Bx and post-ADT RP samples, we performed hierarchical clustering on the median-centered log-normalized counts of the PCS subtype signature genes on 20 specimens in our cohort. Interestingly, we found that the clustering of the subtype signature genes again segregated the high and low impact groups (Supplementary Figure S2), consistent with the clustering of differentially expressed genes. We evaluated whether the expression of the subtype genes in tumors before ADT would inform the relative changes in the subtype signature observed after ADT (Figure 2). Specifically, we calculated the fraction of PCS1, PCS2, and PCS3 genes that were overexpressed in every high impact and low impact sample. We considered a gene to be overexpressed if it was greater than two-fold above the median expression of that gene for all samples. We evaluated the changes in gene expression for each subtype before and after ADT. Before ADT, there was no obvious segregation of high impact and low impact pre-ADT Bx samples. After ADT, all post-ADT RP samples displayed a common decrease in the AR-dependent, PCS2 gene expression, and a similar increase in the PCS3 gene expression, but there was a striking divergence in the expression of the aggressive PCS1 signature (Figure 2A). We found that after ADT treatment, the transcriptional signature indicative of the more aggressive, ADT-resistant, androgen receptor-independent subtype (PCS1) was not only retained, but also significantly increased in the high impact group (*p*-value = 4.66 × 10^−3^ Mann–Whitney test). On the other hand, the low impact group exhibited a relative loss of the PCS1 signature, and only the proportion of genes characteristic of the AR-suppressed PCS3 increased (Figure 2B). Finally, the percent of PCS1 genes expressed in the high impact group was significantly higher than the percentage of these genes in the low impact group (*p*-value = 1.04 × 10^−3^ Mann–Whitney test). Taken together, these data suggest a relative shift in the subtype makeup that correlates with the differential intensity in the transcriptional reaction to ADT.

### 2.2. Identifying Transcription Factors (TFs) Enriched for Unique Targets in the High Impact Network

To elucidate putative transcriptional regulators associated with the transcriptional changes unique to the high impact group, we ascertained the regulatory networks. To accomplish this, we utilized the PANDA algorithm [18] that integrates RNA expression data, protein-protein interaction data and DNA binding motif data to reverse engineer the transcriptional networks. We used PANDA to integrate the protein-protein interaction data from the human protein reference database and experimentally validated the interactions among known cancer-associated drivers and tumor suppressors [21,22], DNA binding motif data found within H3K27ac and DNase1 hypersensitivity regions [23], and RNA-Seq data from 16 Post-ADT RPs. To find TFs that putatively regulated the differential transcriptional response to ADT, as opposed to simply before and after ADT, we focused our analysis on the differences between the high impact and low impact networks. We converted the PANDA z-score normalized edge weights to unique interaction probabilities that estimate how likely a transcription factor regulates a given gene, and identified the transcription factors that were enriched for the gene targets in the high impact network as compared to the low impact network using the cumulative distribution function, (termed Key TFs) [18]. We identified 394 out of 725 Key TFs that were significantly enriched for unique targets in the high impact network, as compared to that of the low impact network using the hypergeometric distribution and Bonferroni correction for multiple testing with a critical *p*-value of 0.05 (*p* < 7.85 × 10^−5^), as performed by Glass et al. [19]. Notably, AR and multiple transcription factors extensively reported to be involved in prostate cancer aggressiveness, such as ETV5 and ETV1 [16] were identified as Key TFs with increased transcriptional targets, supporting the biological relevance of our analysis. Moreover, the transcription factors involved in epithelial to mesenchymal transition (EMT), a critical first step in the metastatic cascade, such as SNAI1, SMAD2/3/4, were also predicted to be significantly associated with the high impact group. Interestingly, the pro-EMT factor SNAI1 has been observed to be upregulated after inhibition of AR signaling via ADT [24].

### 2.3. Finding the Transcription Factor Coordinated Groups (TCFGs) in the High Impact Network

**Figure 3:**
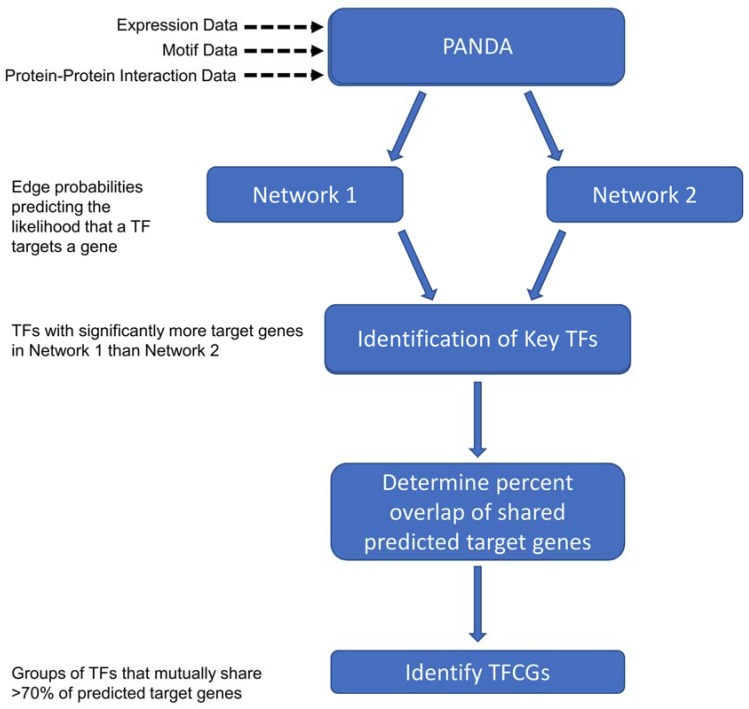
A flowchart depicting the how transcription factor coordinated groups (TFCGs) were identified. Expression-, motif-, and protein interaction data were used as inputs to PANDA. This was run twice using two independent expression datasets (e.g., high- and low impact expression data) to generate networks. Post-processing of the PANDA output (refer to methods) yielded edge probabilities representing the likelihood that a transcription factor targets a given gene. Next, Key TFs were found based on the criteria that a TF gains a significant number of predicted target genes in one network versus another. After determining the percentage overlap of shared predicted target genes (refer to methods), TFCGs were ascertained as groups of Key TFs that share >70% of the predicted target genes.

**Figure 4:**
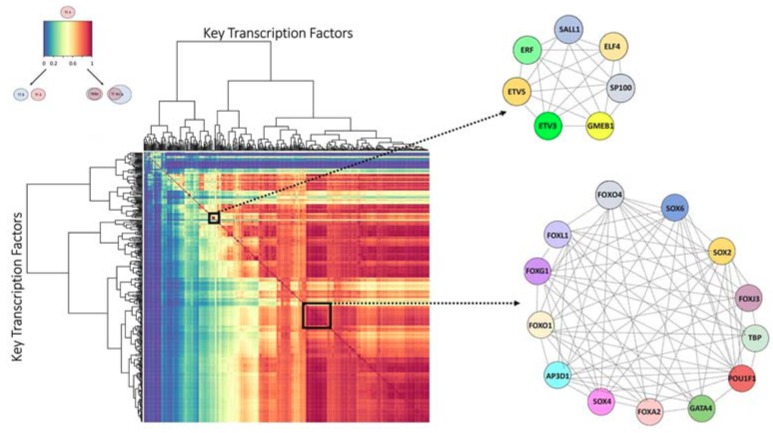
The identification of TFCGs in the high impact group network. The heatmap displays the hierarchical clustering of putative gene target percentage overlap of one Key TF as compared to all others. The dark blue to dark red color gradient denotes the degree of shared target overlap. Because the degree of target overlap between a pair of Key TFs may be non-reciprocal, the dendrograms are ordered based on mutual relationships and are oriented identically on the x- and y-axis. The diagonal represents a Key TF compared to itself. Only reciprocal relationships between groups of Key TFs were considered TFCGs (white boxes demarcate two representative TFCGs as symmetrical squares on the diagonal). Beside the heatmap are two representative TFCG schematics depicting a TFCG containing Key TFs that reciprocally share >70% of their predicted target genes with each other.

Next, we identified the transcription factors that might function in a coordinated fashion by determining the groups of Key TFs that were predicted to regulate the same target genes. We hypothesized that these transcription factor groups that are uniquely enriched in the high impact network, and that were predicted to collaboratively regulate the same genes, might provide insights into the biology of this set of patients with pronounced transcriptional responses to ADT. For each Key TF, we calculated the percentage of target genes that overlapped with the target genes of every other Key TF. We then performed a hierarchical clustering of the pairwise percent overlaps to find TFCGs in which all the Key TF members mutually shared at least 70% of their target genes (Figure 3 and Figure 4). We found 34 TFCGs in this analysis, some of which contained both known oncogenic factors with other factors not previously associated with prostate cancer (Supplementary Table S5). Moreover, within some of these groups were transcription factors with well-described associations, such as AR and FOXA1 (TFCG 33 Supplementary Table S5).

### 2.4. Comparison of the Metastatic PCS1 Network and High Impact Group Network Reveals Common TFCGs

We next investigated whether the high impact transcription factor relationships that emerged early after ADT exposure maintained associations in metastatic tumors. These overlapping TFCGs (oTFCG) may represent important transcription factors that, in a concerted fashion, continue to be active in driving metastasis. To do this, we leveraged a large dataset of over 800 patients compiled from multiple publicly available cohorts that were normalized and subtyped by You et al. [17] to use as the RNA expression data input to PANDA. These cohorts comprised of 2790 gene expression profiles including primary tumors, and metastatic or CRPC tumors [17]. Primary tumors profiles were collected from cohorts including The Cancer Genome Atlas (TCGA). Data from metastatic tumors were derived from the Stand Up To Cancer/Prostate Cancer Foundation Dream Team cohort (SU2C). Our observations suggested an ADT mediated selection against the PCS2 subtype genes, and a relative enrichment of the aggressive PCS1 signature in primary tumors of the high impact group. We hypothesized that there were overlapping TFCGs that were associated with analogous changes in the subtype (the loss of PCS2 and the gain of PCS1 genes) that were also unique to metastatic tumors. Consequently, we elected to find oTFCGs enriched in metastatic tumors of the PCS1 subtype (Met.PCS1) as compared to PCS2 primary tumors.

We first determined the Key TFs that had a significant enrichment of unique targets in the Met.PCS1 network (*p* < 2.19 × 10^−4^) and identified TFCGs (Supplementary Tables S6 and S7). We found that more than 80% of the Met.PCS1 Key TFCGs were also exclusively associated in the high impact group network. We identified 33 TFCGs enriched in both the networks (Table 1) and defined an “overlapping TFCG” (oTFCG) as a TFCG in the Met.PCS1 network that shared at least two Key TFs with a TFCG in the high impact group network. We found groups that contained within them known associations among the transcription factors associated with prostate cancer-relevant signaling pathways, such as JUN and FOS, both member of the MAPK signaling pathway (oTFCG3, Table 1). We also identified groups that contained validated prostate cancer oncogenes with novel associations, such as SOX4, a prostate cancer transforming oncogene, and FOXA2 and GATA4, known pioneer factors (oTFCG13, Table 1) [25]. 

Using independent expression datasets, we identified the common exclusive transcription factor relationships significantly associated with the loss of the PCS2 signal and the retention of the PCS1 signal in the high impact group, and with PCS1 metastatic tumors. Moreover, these transcription factors were predicted to exclusively associate with each other based on sharing distinct sets of target genes in the high impact and Met.PCS1 networks. In some cases, the transcription factors within oTFCGs collectively gained or lost other transcription factors between networks. For example, one TFCG comprised of ERF-ETV5-ETV3-ELF4, gained HIF1A, a well-characterized transcription factor involved in metastasis, in the Met.PCS1 network. The addition or loss of transcription factors from an oTFCG may inform the observed changes in predicted gene targets and suggest an evolving role of these factors in cancer progression. Thus, we identified TFCGs that appear to be functioning in a coordinated fashion to achieve changes in gene expression in two distinct phases of prostate cancer progression. Taken together, these data suggest a concerted condition-dependent re-localization that maintained interactions of these transcription factors during metastasis.

## 3. Discussion

The heterogeneous nature of prostate cancer tumors requires complex and dynamic changes in transcriptional networks to adapt to the various stages of cancer progression. Currently, the regulatory mechanisms that drive these fluctuations in expression profiles throughout the progression are not well understood. ADT is used as one component of treatment for intermediate and advanced prostate cancer. At present, it is difficult to assess the transcriptional changes that are direct consequences of ADT, as the current guidelines discourage the use of neoadjuvant ADT with radical prostatectomy [34]. Our cohort is novel, unique, and unusual in that all but one of the patients received neoadjuvant ADT alone, and subsequent radical prostatectomy, allowing us to interrogate the direct effects of ADT using patient-matched tissues on transcriptional programs. 

A complete understanding of the transcriptional pathways that characterize ADT response and subsequent metastatic progression is still unclear. Previous studies have sought to find molecular mechanisms associated with prostate cancer progression, but these analyses typically rely on expression profiling alone to predict putative upstream regulators [35]. We applied the PANDA [18] algorithm to curate and integrate the expression, protein-protein interaction, and DNA binding data in order to predict the transcription factor-gene target interactions. We utilized these networks to identify the putative cooperative and collaborative transcription factor groups that are most associated with pronounced transcriptional response to ADT, retention of aggressive subtype signatures, and the development of metastatic disease. By employing a top-down approach integrating large, independent datasets, we have predicted and prioritized the transcription factor groups that could serve as critical upstream regulators of ADT response and metastasis. 

Differential expression analysis comparing Pre-ADT Bxs and Post-ADT RPs yielded 190 significantly differentially expressed genes. IPA analysis identified multiple therapeutic agents as putative upstream regulators of these genes. These were largely associated with androgen receptor (AR) signaling (Supplementary Table S2). Specifically, genes regulated by dihydrotestosterone (androgen), and metribolone (R1881), a widely used AR agonist that has been shown to increase the expression of AR target genes [36], were both inhibited in our dataset, as would be expected from ADT. Intriguingly, despite relatively short ADT exposure times, we identified divergent transcriptional responses segregating the high and low impact groups. Accordingly, KLK3 (PSA) expression was significantly lower in the high impact group than in the low impact group, underscoring the biological relevance of the differential transcriptional responses between the two groups. This may also indicate the initial AR signaling inhibition in response to ADT before the eventual reactivation of this signaling pathway [8,37]. IPA analysis also showed that the beta-estradiol (estrogen) targets were activated and the targets of U0126, a MAPK kinase pathway inhibitor, were inhibited in response to ADT, suggesting a bypass or compensatory PDGF-MAPK and estrogen signaling. Concordantly, Hu et al. found that AR expression is associated with a favorable outcome in ER^+^ breast cancers. On the other hand, they also found that AR expression in ER-negative TNBC breast cancers was significantly associated with increased mortality, as compared with AR-negative, ER-negative TNBC tumors (multivariate hazard ratio (model 3), 1.83; 95 percent confidence interval, 1.11 to 3.01; *p* = 0.02) [38]. Taken together with our observations, these data suggest a compensatory relationship between AR and estrogen signaling in both prostate cancer and triple negative breast cancers. While the IPA analysis provided initial insights into the initial transcriptional responses to ADT, among well-established signaling networks, we were interested in identifying novel transcriptional relationships in this setting.

We characterized the transcriptional responses in our cohort using a previously developed subtyping scheme developed by You et al. [17]. These PCS subtypes were developed by integrating a priori defined prostate cancer-relevant signaling pathways, genetic and genomic alterations, and other biological characteristics of aggressive prostate cancer such as stemness and cell proliferation [17]. The PCS1 subtype is the most aggressive of the three subtypes, with a poorer prognosis, shorter metastasis-free survival, and metastatic CRPC [17]. This aggressive subtype is enriched for the AR-variant pathway genes and it is also associated with enzalutamide-resistance [17,39] and metastatic-CRPC [15]. The PCS2 subtype is enriched for AR-signaling genes, and was found to be sensitive to enzalutamide [17]. The PCS3 subtype exhibits a low expression of AR-signaling genes [17,40] and it is associated with gene signatures enriched in basal cells [40]. 

In contrast to the similar transcriptional changes in the PCS2 and PCS3 signatures, we observed a striking difference in the percentage of PCS1 genes expressed in the high and low impact groups following ADT. The high impact group exhibited not only a retention of this signal, but in many cases, an increase in expression of the PCS1 genes, while the low impact group tended to lose expression of these genes (Figure 2). These data suggest that despite similar ADT exposure times for all patients, only the tumors of the high impact group were associated with a more aggressive subtype. We speculate that the high impact group tumors could resist an ADT mediated selection against aggressive components, or clonal populations by activating transcriptional programs that adapt to the inhibition of AR signaling, while those of the low impact group were more sensitive to the effects of ADT treatment [17].

Many studies utilize expression profiling alone to elucidate critical gene signatures important to prostate cancer [35]. We aimed to find the common transcription factor combinatorial relationships that putatively regulate the transcriptional programs associated with ADT response and metastasis. A limitation of our study is that we do not directly address or characterize patients who present with metastases at diagnosis and are still hormone-naïve. However, the TFCG associations that we describe here may serve as critical upstream hubs for patients who have undergone ADT treatment. A Key TF might gain putative target genes in the high impact group network because it is either upregulated or has increased accessibility for target genes due to changes in the local epigenetic landscape [41]. Therefore, we did not require that a Key TF exhibit a significant increase in expression. These groups contained both known and undescribed relationships. For example, TFCG33 (Supplementary Table S5) contained AR and FOXA1, a well-studied AR collaborating factor. Interestingly, this TFCG also contained STAT3, which was recently shown to be co-regulated with AR by Janus Kinase/IL-6 [42]. The strength of our analysis relies on the integration of context-dependent transcriptional data and validated protein-protein interactions with DNA motif data to infer condition-specific TFCGs. 

Next, we sought to ascertain whether associations identified among transcription factors in the high impact group after ADT were also present in metastatic samples. We integrated gene expression data from a large cohort of unmatched primary and metastatic samples (*n* > 800) subtyped by You et al. [17] with our own analysis, and interrogated the transcriptional differences between primary PCS2 tumors and PCS1 metastases. TFCGs, common between the high impact group and metastatic lesions of PCS1, could shed light on potential drug targets and actionable pathways that can be inhibited earlier in prostate cancer development. Intriguingly, by using independent gene expression datasets, we found that more than 80% of TFCGs in the Met.PCS1 network were also present in the high impact network. These observations could reflect a possible ADT-mediated clonal selection of aggressive cancer cells (reviewed in Reference [43]).

Considerable research has been devoted to understanding how the collaborative transcription factor relationships and interdependencies confer a precise temporal control of condition-specific changes in transcription (reviewed in Reference [44]). Despite dynamic transcriptional changes that occur in a tumor in response to ADT and during metastatic progression, we identified common TFCGs that emerge early after ADT administration, and remain associated with each other in metastatic tumors, the final stage of cancer progression. The common TFCGs between the high impact group and Met.PCS1 networks can reflect both spatial and temporal interactions. Intriguingly, these relationships remained despite targeting a distinct set of genes between the networks. This possibly reflects a refinement of more robust TFCG associations that are more critical in influencing both a pronounced transcriptional response to ADT, retention of aggressive subtype pathways, and metastatic progression. 

ADT resistance is associated with AR splice variants (e.g., AR-V7) [45], genomic amplification of AR [46], and mutations to the AR ligand binding domain [47] that maintain transcriptional activity in a low androgen environment. Interestingly, AR separates from a large TFCG in the high impact group network, and associates with C/EBPβ, C/EBPδ, C/EBPγ, and C/EBPε in the Met.PCS1 network. The C/EBP family of transcription factors is associated with mesenchymal gene signatures and aggressive diseases in a variety of tumors including glioblastomas [48,49,50], esophageal squamous cell carcinoma [51], urothelial carcinoma [52], and hepatocellular carcinoma [53]. Additionally, C/EBPβ promotes oncogene-induced senescence, facilitating tumor progression and chemotherapy resistance after androgen deprivation [54]. This may reflect distinct transcriptional programs for AR to both evade the effects of ADT and promote metastases, and sheds light on the dynamic interactions of AR to maintain signaling throughout the prostate cancer progression, despite changes in androgen availability.

The SOX4-FOXA2-GATA4 oTFCG (oTFCG13 Table 1) is also of interest for multiple reasons. We have shown that deletion of SOX4 in vivo can inhibit prostate cancer progression [55] and that the knockdown of SOX4 [25] can induce apoptosis in prostate cancer cells. Moreover, in pancreatic cancer, SOX, FOX, and GATA factors may cooperate to drive metastases [56].

Importantly, the oTFCGs that contain transcription factors regulated by distinct signaling pathways suggest not only a possible compensatory activation after ADT, but also suggest a maintained activity of these pathways throughout the progression to metastasis. FOS, FOSB, FOSL1, JUN, JUND, and JUNB, all members of the MAPK signaling pathway, were associated with each other in both high impact and Met.PCS1 networks, and FOS and FOSB were also significantly overexpressed after ADT. Notably, IPA analysis informed the biological relevance of this oTFCG as it showed that targets of U0126, a MAPK kinase pathway inhibitor, were inhibited in response to ADT. Combining our observations of decreased KLK3 expression with the IPA analysis indicating an inhibition of AR-signaling, these data suggest a bypass or compensatory PDGF-MAPK signaling after ADT. This is consistent with previous reports of increased phospho-MAPK levels enriched in tumor tissues of patients who have undergone ADT [57]. Moreover, it has recently been shown that AR-null prostate cancers that do not undergo neuroendocrine differentiation, or ‘double negative’ metastatic prostate cancers have sustained FGF-MAPK signaling and that these cancers are sensitive to MEK and ERK1/2 inhibition in vitro and in vivo [58]. Thus, both the IPA analysis and our finding that these factors maintain associations in the high impact group, and metastatic tumors suggest that combination therapies that include ER, MEK, JNK, and/or ERK inhibitors may provide some benefit to patients undergoing ADT.

## 4. Materials and Methods

### 4.1. Tissue Specimens

Patient tissue specimens and associated clinical data were selected from the prostate cancer biobank of the Centre hospitalier de l’Université de Montréal research center (CRCHUM). All patients signed an informed consent form to participate in the biobank and the Comité d’éthique à la recherche of the CRCHUM approved the study. We selected patients with matched pre- and post-androgen deprivation therapy specimens of biopsy and RP performed at the CHUM between 1993 and 2012. Following the review of the hematoxylin/eosin (H&E) -stained slides by a genitourinary pathologist, the tumor areas were identified. Biopsies and prostatectomies pathology reports were used to establish the localization of the tumors. If relevant, (i.e., no effects of hormone therapy), histologies were also compared. Of note, the tumor locations could not be confirmed for every patient. For RP, the whole case was reviewed to identify the index tumor or, when it could be identified from the biopsy report, the nodule from which the biopsy was taken. The corresponding area on the FFPE tumor blocks were extracted using a 0.6 mm diameter tissue arrayer needle (TMArrayer; Pathology Devices, Inc., Westminster, MD, USA) and transferred into a 1.5 mL plastic tube prior to extraction. The FFPE biopsies were macrodissected prior to RNA preparation to exclude benign tissues. 

### 4.2. RNA-Sequencing and Differential Gene Expression Analysis of Pre/Post ADT Patient Samples

The total RNA from 40 matched FFPE specimens from Pre-ADT core biopsies and Post-ADT radical prostatectomies were sequenced using Illumina’s TruSeq RNA Access Library Prep kit. Sequence alignment and gene level expression quantifications were obtained using the STAR read aligner to map them to the hg38 reference genome [59,60,61]. Differential gene expression analysis was performed with the edgeR Bioconductor package in R. The final statistical model included corrections for sequencing the batch effects and sequence coverage. Datasets can be accessed in the NCBI GEO and SRA databases (accession No. GSE111177).

### 4.3. Constructing Transcriptional Networks Using PANDA

To infer interactions between transcription factors and gene targets, we used three datatypes as inputs to PANDA: co-transcriptional expression, protein-protein interaction (PPI), and transcription factor binding site (TFBS) motif data, described in detail below [18]. Briefly, we used PANDA to construct a network for each condition (e.g., pre-, post-ADT) using constant PPI and protein-gene datasets, but condition-specific gene expression data. PANDA was run with default parameters (alpha = 0.2, hamming distance = 1 × 10^−5^) using MATLAB. Transcription factors that were absent in either the PPI or expression data (normalized DESeq counts < 1) were removed. 

### 4.4. Protein-Protein Interaction Data

Binary (direct interactions between two proteins) PPI data were obtained from the human protein reference database (HPRD) (http://www.hprd.org), and OncoPPI [21]. HPRD contains interactions that are derived from experimental evidence from the literature [22], and OncoPPI contains interactions from the TR-FRET screening data. 

### 4.5. Motif Data

The regions of H3K27Ac and DNaseI hypersensitivity within the human genome (build hg19) were obtained from the ENCODE data tracks [62] in the UCSC human genome database [63]. We utilized the MATCH software [23] based on the BioBase Knowledge Library 2017.3 TRANSFAC database [64] to identify all vertebrate TFBS in these regions with a minimum core matrix score greater than 0.95. Motif data were further annotated to retrieve the HGNC symbols corresponding to the TRANSFAC transcription factor position weight matrix identifiers. 

### 4.6. Expression Data

Mapped read counts were normalized using the DESeq Bioconductor package. Log-2 transformed normalized counts were centered by the median of all samples in the dataset. For primary and metastatic network construction, we obtained median-centered normalized expression data of publicly available datasets containing more than 800 patient samples that were curated and subtyped by You et al. [17].

Identification of network-specific “Key” transcription factor coordinated groups and gene target expression analysis

To identify the transcriptional relationships that were significantly altered between two conditions, we first compared pairs of networks to identify the interactions between transcription factors and gene target interactions that were unique to one network versus another. Briefly, the program uses a message-passing procedure to estimate the agreement of data types by calculating a similarity score that represents the support that a gene is putatively targeted by a transcription factor. We included TFs that had an expression value count greater than 1 for the DESeq normalized counts [65]. The similarity scores, or edge weights, are the z-scores normalized to allow for the iterative updating of these edge weights across the data types [18]. The PANDA algorithms outputs z-scores that represent the support that a transcription factor targets a gene in a given network. We identified unique interactions by converting the z-scores to “unique interaction probabilities” as described in Glass et al. [19]. Briefly, we used the cumulative distribution function to generate probabilities representing whether an interaction was unique to, and strongly supported in, one network but not in another. We selected the transcription factor–target gene interactions that had a probability greater than 90%. Next, we identified the transcription factors that were significantly enriched for gene targets in one network versus another by employing the hypergeometric distribution and Bonferroni correction for multiple testing with a critical *p*-value of 0.05 (Key TFs). To uncover the transcription factors that share common gene targets in a network, we performed non-reciprocal pairwise comparisons to determine the percent overlap of predicted high confidence target genes shared between two Key TFs followed by the hierarchical clustering of these percentages to reveal putative coordinated groups. We defined a coordinated group as containing Key transcription factors that exclusively share at least 70% of their targets. Finally, we performed hierarchical clustering (Euclidean dissimilarity metric, complete agglomerative clustering) of the shared target gene expression. 

To assess whether we would identify a given number of Key transcription factors by chance alone, we permuted the RNA-seq sample identifiers without replacement. We used the permuted RNA-seq data as one of the inputs for PANDA, and performed analyses to identify the Key transcription factors. The permuted network did not contain any transcription factor–gene target interactions with a probability greater than 90%, demonstrating the significance of the identified pairs in the actual network. 

## 5. Conclusions

Our analysis utilizes a novel, unique dataset from matched patient samples that interrogates the direct effects of ADT, and integrates these data with a large cohort of primary and metastatic tumors to predict the common mechanisms important to the resistance to therapy and the progression to a metastatic disease. We have elucidated the transcription factor relationships that are consistently associated with aggressive ADT transcriptional responses, and metastasis: two distinct, and clinically significant phases of prostate cancer progression. This hypothesis-driving study expands what is known about the important coordinated transcription factor activities that regulate prostate cancer aggressiveness, metastasis, and the development of androgen-resistance. 

## Figures and Tables

**Table 1:** Thirty-three overlapping TFCGs (oTFCGs) between the high impact ADT group and Met.PCS1 networks. Annotations are derived from the KEGG pathway database and/or the primary literature.

Group Name	Transcription Factors	Kegg Pathway Annotation and/or Reference
oTFCG1	NR2F2-SMAD9-PAX2-TAL1-ELK4-ELK3-KLF12-ETV6-SMAD7-MAFA-TCF7L2-ETV4-SREBF2-GATA3-MYBL2-MYB-YBX1-ERG-FLI1-RFX1-SREBF1-HSF4-ZEB1-GABPA-ELF1-ELF5	Transcriptional misregulation in cancer; TGF-β signaling
oTFCG2	MYF5-TCF4-MYOG-TCF12-NR2C2-NF1A-SMAD5-PAX4-ELK1-SPIB-MYOD1-TCF3-GATA1-NFIX-KLF4-PURA-KLF6-GEN1-E2F3-TFDP1-GTF2I-HIC1-WT1-E2F4	Pathways regulating pluripotency of stem cells
oTFCG3	JUN-SOX10-SOX18-JUND-JUNB-SMAD3-FOS-RXRA-BRCA1-SMAD2-NR3C1-ETS2-GATA2-YY1-TCF7L1-FOSL2-FOSB-FOSL1	MAPK signaling; osteoclast differentiation; IL-17 signaling pathway; Wnt signaling; TGF-β signaling
oTFCG4	NFKB1-MTF1-ZIC3-TFCP2-ZBTB7A-MZF1-BCL6B-SP4-SP3-ZIC1-SP2-TP73-TP63	MicroRNAs in cancer
oTFCG5	HES1-IKZF1-TFAP2C-PAX8-RUNX3-ETV7-THAP1	Pathways in Cancer
oTFCG6	HSF1-HNF1A-SOX17-FOXM1-IRF4-NKX2-5	Wnt Signaling
oTFCG7	NR1H3-RARB-NR1I2-RARG-NR1H2-NR1I3	Insulin resistance; Small cell lung cancer; Non-small cell lung cancer
oTFCG8	GATA5-SRY-SOX8-POU2F1-CUX1	
oTFCG9	GATA6-FOXA1-POU3F3-FOXD3	EMT in pancreatic cancer [26]
oTFCG10	EGR1-KLF13-EGR2-HIC2	GnRH signaling; Human T-cell leukemia virus 1 infection
oTFCG11	ERF-ETV5-ETV3-ELF4	Transcriptional misregulation in cancer; Prostate Cancer
oTFCG12	EP300-SPI1-SMAD4-E2F1	Pathways in Cancer; Human T-cell leukemia virus 1 infection; TGF-beta signaling; Prostate Cancer; Wnt signaling; Cell cycle
oTFCG13	SOX4-FOXA2-GATA4	Prostate cancer oncogene [25]
oTFCG14	E2F7-E2F5-E2F2	Gastric Cancer; Prostate Cancer; Bladder Cancer
oTFCG15	HOXB2-PRRX2-PDX1	TGF-beta signaling induced invasion in breast cancer [27]
oTFCG16	ARNT-TFAP2A-TFAP2B	Cushing Syndrome; HIF-1 signaling; Renal Cell Carcinoma
oTFCG17	NFYC-NFYA-NFYB	Antigen processing and presentation
oTFCG18	MAFK-CEBPG-CEBPE	Transcriptional misregulation in cancer; Acute myeloid leukemia
oTFCG19	MAZ-ARHGEF7-CD40	Regulation of actin cytoskeleton
oTFCG20	MAX-EGR3-ZIC2	C-type lectin receptor signaling; Small cell lung cancer; Transcriptional misregulation in cancer; MAPK signaling
oTFCG21	GLI3-GLI2	Hedgehog signaling; Basal Cell Carcinoma; Hippo signaling
oTFCG22	ZBTB33-PLAGL1	Metastasis and TGF-β signaling in triple negative breast cancer [28]; Cell cycle [29]
oTFCG23	HDAC1-UBP1	Epigenetic reprogramming in cancer (HDAC) [30]
oTFCG24	FOXL1-TBP	Huntington disease; Basal transcription factors
oTFCG25	KLF2-RREB1	MAPK Signaling; FOXO signaling
oTFCG26	USF2-USF1	Inhibition of cell cycle [31]
oTFCG27	CEBPB-CEBPD	TNF Signaling pathway; Transcriptional misregulation in cancer
oTFCG28	CHURC1-TEAD2	EMT in breast cancer [32]
oTFCG29	ETV1-HIF1A	HIF1-signaling; Angiogenesis; Prostate cancer invasion [33]
oTFCG30	ATM-GTF2IRD1	FoxO signaling; Cell cycle; NF-kappa β signaling
oTFCG31	MYC-RXRB	Gastric Cancer; Thyroid hormone signaling; Small cell lung cancer; PPAR signaling
oTFCG32	SP1-TP53	Endocrine resistance; Huntington disease; Breast cancer; Transcriptional misregulation in cancer; Endocrine resistance
oTFCG33	NR4A2-TFAP4	MAPK Signaling; osteoclast differentiation; IL-17 signaling; Wnt signaling; TGF-β signaling

## Supplementary Materials

The following are available online at http://www.mdpi.com/2072-6694/10/10/379/s1, Supplementary Table S1, Clinical and pathological patient data from 20 patients; Supplementary Table S2, Significantly differentially expressed genes identified using edgeR analysis of pre-ADT Bxs and post-ADT RPs; Supplementary Table S3, Ingenuity Pathway Analysis suggests upstream regulators associated with significantly differentially expressed genes; Supplementary Table S4, Key TFs in the high impact network; Supplementary Table S5, TFCGs in the high impact network; Supplementary Table S6, Key TFs in the Met.PCS1 network; Supplementary Table S7, TFCGs in the Met.PCS1 network; Supplementary Figure S1, The high impact group displays a significant decrease in “downregulated” gene group expression, and significant increase in “upregulated” gene group expression, as compared to the pre-ADT Bx and low impact group samples; Supplementary Figure S2, Hierarchical clustering of PCS genes of 20 matched pre-ADT Bxs and post-ADT RPs again segregates post-ADT RP samples.
