# Supplementary material for: Identification of the Transcription Factor Relationships Associated with Androgen Deprivation Therapy Response and Metastatic Progression in Prostate Cancer
Source: Cancers (Basel). 2018 Oct 11;10(10):379. doi: 10.3390/cancers10100379 (PMC6210624; doi:10.3390/cancers10100379)
Supplement: Supplementary file 1 [file cancers-10-00379-s001.zip › cancers-357908-suppl.-proof-/cancers-357908-suppl.-proof-.docx]

**Supplemental Materials:** **Identification of the Transcription Factor Relationships Associated with Androgen Deprivation Therapy Response and Metastatic Progression in Prostate Cancer**

Nitya V. Sharma, Kathryn L. Pellegrini, Veronique Ouellet, Felipe O. Giuste, Selvi Ramalingam, Kenneth Watanabe, Eloise Adam-Granger, Lucresse Fossouo, Sungyong You, Michael R. Freeman, Paula Vertino, Karen Conneely, Adeboye O. Osunkoya, Dominique Trudel, Anne-Marie Mes-Masson, John A. Petros, Fred Saad and Carlos S. Moreno

**Table S1.** Clinical and pathological patient data from 20 patients.

**Table S2.** Significantly differentially expressed genes identified using edgeR analysis of pre-ADT Bxs and post-ADT RPs. Abbreviations FC, fold change; CPM, counts per million; FDR, false discovery rate.

**Table S3.** Ingenuity Pathway Analysis suggests upstream regulators associated with significantly differentially expressed genes. Predicted upstream chemical agent regulators suggest inhibition of androgen driven genes, and increase in estrogen and PDGF-MAPK signaling.

**Table S4.** Key TFs in the high impact network. 394 Key TFs with significant enrichment for predicted genes in high impact group as compared to low impact group. The TF edge title refers to the number of putative gene targets that a given TF is predicted to regulate. *p*-values are generated via the hypergeometric distribution and Bonferroni correction for multiple testing with a critical *p*-value of 0.05.

**Table S5.** TFCGs in the high impact network. 34 TFCGs were identified in the high impact network as defined as sharing >70% of predicted target genes.

**Table S6.** Key TFs in the Met.PCS1 network. 228 Key TFs with significant enrichment for predicted genes in Met.PCS1 specimens as compared to Prim.PCS2 specimens. The TF edge title refers to the number of putative gene targets that a given TF is predicted to regulate. *p*-values are generated via the hypergeometric distribution and Bonferroni correction for multiple testing with a critical *p*-value of 0.05.

**Table S7.** TFCGs in the Met.PCS1 network. 27 TFCGs were identified in the Met.PCS1 network as defined as sharing >70% of predicted target genes.


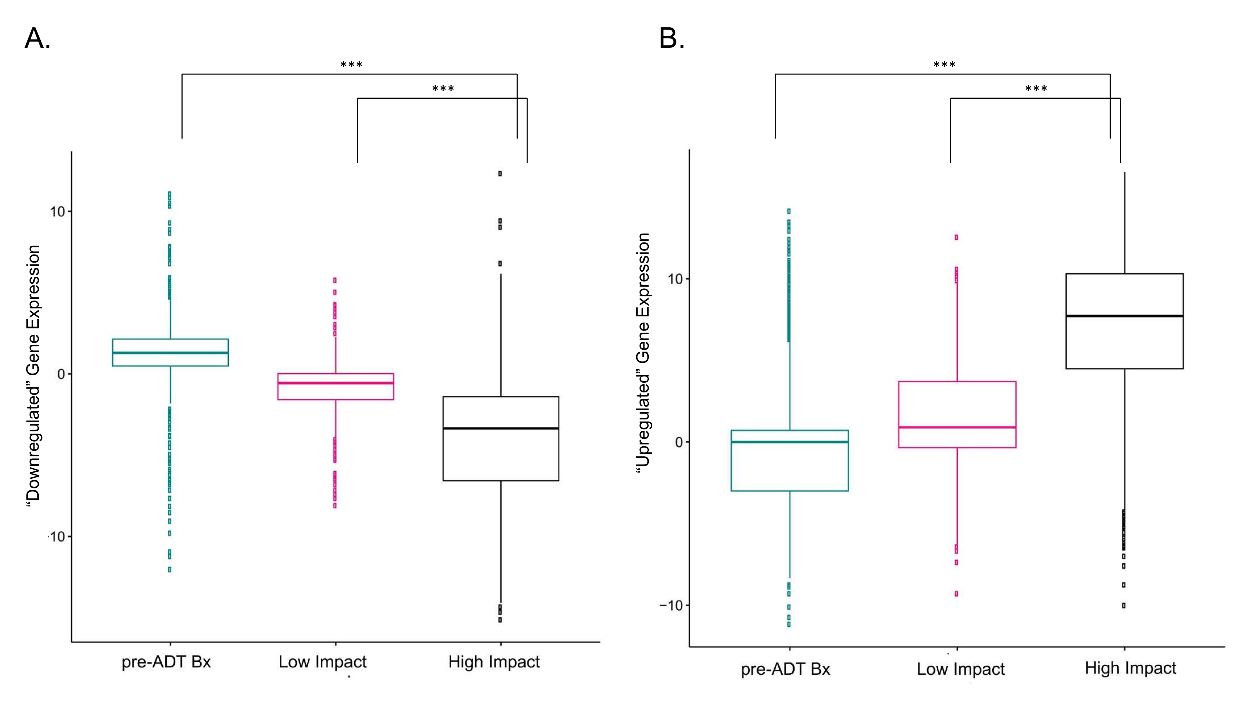


**Figure S1.** The high impact group displays a significant decrease in “downregulated” gene group expression, and significant increase in “upregulated” gene group expression, as compared to the pre-ADT Bx and low impact group samples. Boxplot of relative gene expressions for “upregulated” and “downregulated” significantly differentially expressed genes in pre-ADT Bx, low impact and high impact samples. The high impact group samples have a significantly higher mean expression of “upregulated” genes (one-way ANOVA and Tukey Honest Significant Differences *p*-value = 2.14 × 10^−8^), and a significantly lower mean expression of “downregulated” genes (one-way ANOVA and Tukey Honest Significant Differences *p*-value = 3.59 × 10^−11^), than the pre-ADT Bx and low impact group samples.


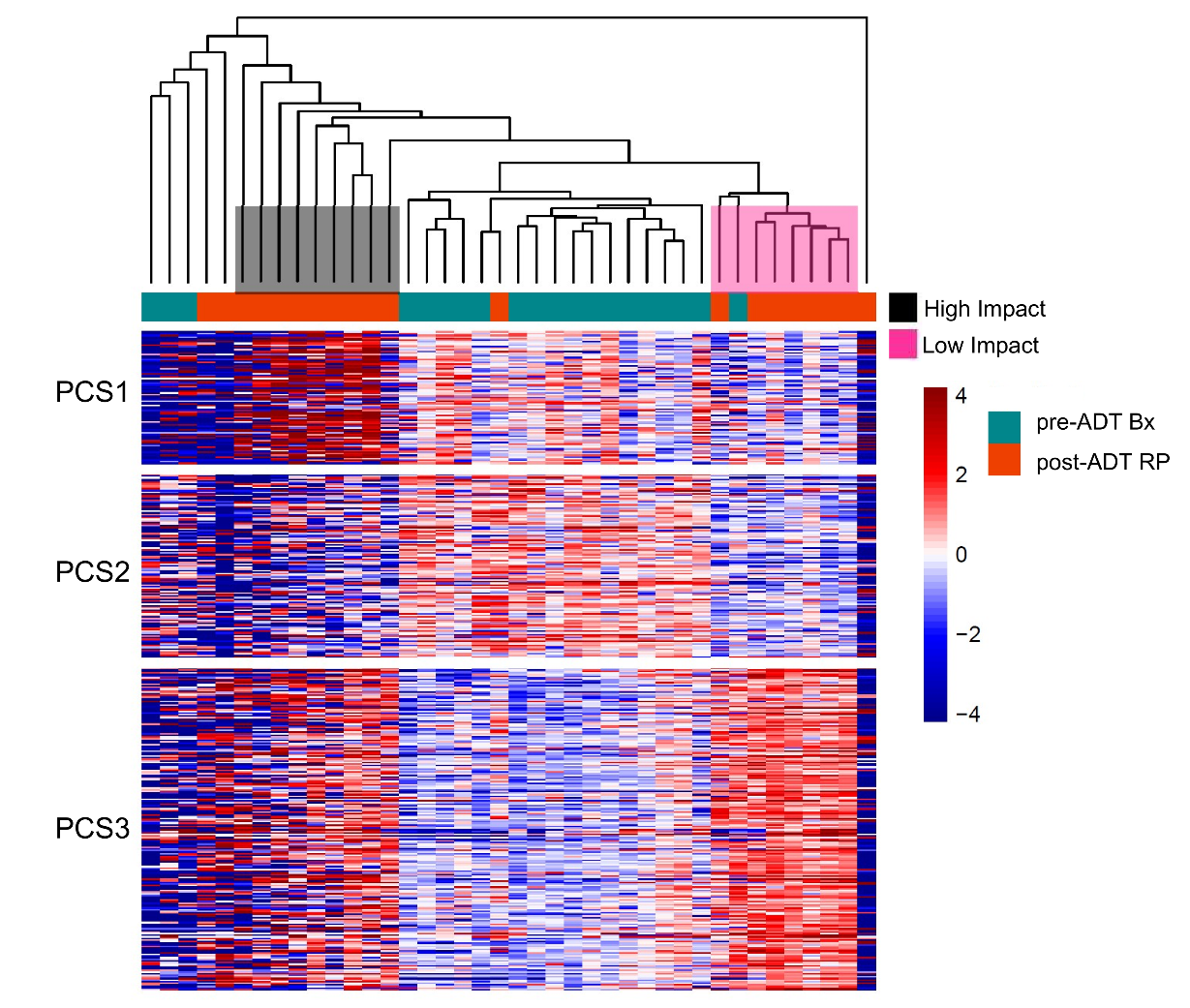


**Figure S2.** Hierarchical clustering of PCS genes of 20 matched pre-ADT Bxs and post-ADT RPs again segregates post-ADT RP samples. Hierarchical clustering of median-centered log2-normalized counts reveals two distinct groups. High impact group (black) predominantly express PCS1 and PCS3 subtype genes. Low impact group (magenta) predominantly express PCS3 genes.


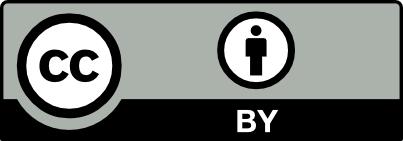
© 2018 by the authors. Licensee MDPI, Basel, Switzerland. This article is an open access article distributed under the terms and conditions of the Creative Commons Attribution (CC BY) license (http://creativecommons.org/licenses/by/4.0/).
